# Influence of Pore Size and Fatigue Loading on NaCl Transport Properties in C-S-H Nanopores: A Molecular Dynamics Simulation

**DOI:** 10.3390/ma13030700

**Published:** 2020-02-04

**Authors:** Qingyu Cao, Yidong Xu, Jianke Fang, Yufeng Song, Yao Wang, Weiguo You

**Affiliations:** 1 Central Research Institute of Building and Construction, MCC Group, Beijing 100088, China; achero@126.com; 2 Engineering Research Center for Water Engineering Safety and Disease Prevention and Control (MWR), Changjiang River Scientific Research Institute, Wuhan 430010, China; 3 Ningbo Institute of Technology, Zhejiang University, Ningbo 315100, China; fjkleo1993@163.com (J.F.); songyufeng567@163.com (Y.S.); wy903961287@sina.com (Y.W.); youasxp@163.com (W.Y.); 4 College of Civil Engineering and Architecture, Zhejiang University, Hangzhou 310017, China

**Keywords:** C-S-H, diffusion coefficient, pore diameter, fatigue loading, molecular dynamics simulation

## Abstract

The transport properties of chloride ions in cement-based materials are one of the major deterioration mechanisms for reinforced concrete (RC) structures. This paper investigates the influence of pore size and fatigue loading on the transport properties of NaCl in C-S-H nanopores using molecular dynamics (MD) simulations. Molecular models of C-S-H, NaCl solution, and C-S-H nanopores with different pore diameters are established on a microscopic scale. The distribution of the chloride ion diffusion rate and the diffusion coefficient of each particle are obtained by statistically calculating the variation of atomic displacement with time. The results indicate that the chloride ion diffusion rate perpendicular to C-S-H nanopores under fatigue loading is 4 times faster than that without fatigue loading. Moreover, the diffusion coefficient of water molecules and chloride ions in C-S-H nanopores increases under fatigue loading compared with those without fatigue loading. The diffusion coefficient of water molecules in C-S-H nanopores with a pore size of 3 nm obtained from the MD simulation is 1.794 × 10^−9^ m^2^/s, which is slightly lower than that obtained from the experiment.

## 1. Introduction

The deterioration of reinforced concrete structures in coastal areas is mainly caused by chloride ions [1,2]. Considerable research has been carried out on corrosion properties in structural concrete on a macro level [3,4,5]. Nemecek [6] considered the concentration change of chloride ions under diffusion convection and simulated the transport of chloride ions in reinforced concrete models with FEM simulation, which is based on Fick’s first law. Li [7] introduced a multiscale approach of combining both mesoscopic models, including full-graded aggregate and equivalent macroscopic models. Feasibility and relative error were evaluated by analytical deduction and numerical simulation. Carsana [8] investigated the effects of chlorides in raw materials, such as recycled aggregate from salt-contaminated concrete structures, on the durability of concrete. The tested durability-related properties included capillary water absorption, chlorides, and carbonation penetration. Zhang [9] experimentally studied the combination effect of freeze–thaw cycles and chloride attack on concrete damage. Since reinforcement corrosion coupled with sustained load has been recognized as the main issue affecting the durability of reinforced concrete (RC) structures [10], many existing studies have been conducted to investigate the properties of structural concrete under the coupled effect. Li [11] tested the performance of RC beams under coupled sustained loading and reinforcement corrosion. The authors of this study [12] proposed a concrete damage plasticity model to simulate the damage evolution of RC beams under the coupled effect.

Figuring out chloride ion diffusion mechanism in cement-based materials is of vital importance to improve the durability of RC structures in marine environments [13,14]. Many researchers have also investigated the chloride diffusion process in cement-based materials on a micro scale [15,16,17]. Zheng [11] derived the aggregate number function of the circular aggregate, which is based on the probability density function and the cumulative distribution. A Brownian motion simulation for the chloride diffusivity of concrete was conducted, which was verified through two sets of experimental results. He [12] established the relationship between chloride diffusion coefficient pore structure parameters, which was characterized by low-field nuclear magnetic resonance (NMR) spectroscopy. Liu proposed a multi-phase transport model to simulate the ionic (K^+^, Na^+^, Cl^−^, and OH^−^) transport features in concrete composites containing various shaped aggregates. Li [15] established a model that used the concept of double porosity to reflect the influence of pore size distribution on the transport of ionic species in porous materials. The model also considered the effects of ionic exchange between the pores of different sizes and the ionic binding between liquid and solid phases. The model was validated using experimental data obtained from rapid chloride migration tests. Using the molecular dynamics (MD) simulation method, Hou [16] investigated the structural and dynamic properties of water/ions and a tobermorite interface. Li [17] established nanopores of cement-based materials with different widths and tortuosity. The transport process of chloride ions in nanochannels was explored. The results showed that the larger the width of the nanopores, the faster the diffusion rate of chloride ions. The tortuosity of nanopores changes the direction of chloride ion transport, leading to a reduction of the chloride ion diffusion rate. Yang [8] predicted the chloride ionic diffusivity in cement-based microstructures by pore-scale modeling using a modified lattice Boltzmann method. Both the Nernst–Planck equation for ion diffusion and the Poisson equation for electrodynamic effect were fully solved. The results showed that a cement-based microstructure with a smaller pore size and higher negative Zeta potential hindered chloride ion corrosion more effectively.

The abovementioned studies, however, were mainly focused on corrosion properties of RC structure not subjected to fatigue loading, yet the coupled chemo-mechanical process of chloride diffusion was not considered. Furthermore, the application of MD simulation on chloride transport properties in cement-based materials has not been thoroughly discussed, apart from a few exceptions [16,17,18].

The aim of this paper is to reveal the essence of the particle transport properties in C-S-H nanopores under real working conditions. The objectives of this research include (1) establishing molecular models of C-S-H, NaCl solution, and C-S-H nanopores; (2) simulating the transmission process of particles in a NaCl solution in C-S-H nanopores under different working conditions; (3) investigating the effects of pore size and fatigue loading on the NaCl transport properties in C-S-H nanopores; and (4) exploring the distribution of chloride ion diffusion rate and mean square displacement (MSD) of particles in NaCl solution.

## 2. Molecular Dynamics Simulation Theory

Newton’s law of motion is the basic principle of a molecular dynamics simulation, in which the microscopic particles (atoms, molecules, or ions) are treated as Newtonian classic particles and the phase space trajectories of these particles can be calculated at discrete time intervals. The position, velocity, and acceleration of each particle at each time step can be calculated, and the macroscopic properties of the system can be obtained using the statistical mechanics method. The calculation steps of the molecular dynamics simulation are as follows:

(1) Establish the micro-structural system needed for a specific project.
(2) Given the initial position and velocity of each atom in the micro-structural system, the appropriate potential function is adopted to calculate the load on each atom. The following two formulas are the speed and position of atom i at time t, which are the core of the numerical solution equation of molecular dynamics simulation. The correctness of the atom’s numerical solution depends on *U* (potential function).

(1) $$\nu_{i}=\frac{1}{m_{i}}\frac{dU(r)}{dr_{i}}t+v_{i}^{0}$$

(2) $$r_{i}=\frac{1}{2}\frac{1}{m_{i}}\frac{dU(r)}{dr_{i}}t^{2}+v_{i}^{0}t{+r}_{i}^{0}$$

where: *v_i_* is the velocity of atom *i* at time *t*; *r_i_* is the position of atom *i* at time t; *v_i_*^0^ is the initial velocity of atom i; *r_i_*^0^ is the initial position of atom *i*.

(3) Based on the obtained position, velocity, and load of the atom at each time, the atom at the upper position is pushed to a lower energy state in a small-time interval, thus generating a new position and velocity.
(4) The positions and velocities of the atoms are updated in the established micro-structural system. The simulation steps mentioned above are repeated until the properties of the system do not change with time, and the structure of the system reaches a stable state. The trajectories of all atoms in the micro-structural system can be obtained.

## 3. Molecular Dynamics Simulation Details

### 3.1. Molecular Modeling of NaCl Transportation in C-S-H

To investigate the transport characteristics of NaCl in C-S-H nanopores on a micro-scale, a molecular model of C-S-H, NaCl solution, and C-S-H nanopores containing NaCl was established. The modeling details are as follows:

#### 3.1.1. Molecular Modeling of C-S-H

**Figure 1:**
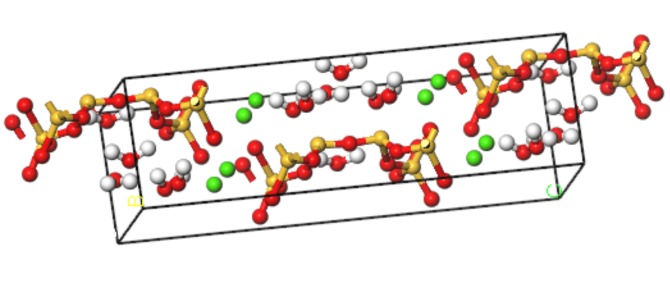
Molecular modeling of C-S-H (yellow balls are Si atoms, green balls are Ca atoms, red balls are O atoms, white balls are H atoms).

The name “tobermorites” includes a number of C-S-H phases differing in their hydration state and sub-cell symmetry [19]. Using the nuclear magnetic resonance (NMR) method, Merlino [20] obtained the microstructure of tobermorite with a layer spacing of 11 Å, which has a structure similar to that of C-S-H. The detailed microstructure parameters are shown in Table 1. A corresponding C-S-H molecular model is established, as shown in Figure 1.

#### 3.1.2. Molecular Modeling of NaCl Solution

**Figure 2:**
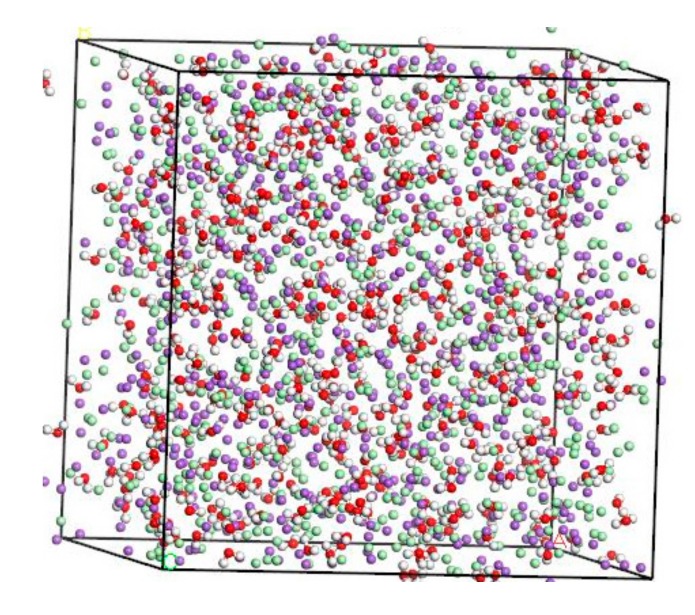
Molecular modeling of NaCl solution (purple balls are Na^+^, light green balls are Cl^−^, red balls are O atoms, white balls are H atoms).

The concentration of NaCl solution used herein is 8%, which is refined to the ratio between Na^+^, Cl^-^, and H_2_O on a micro-scale. According to the calculation result, Na^+^:Cl^−^:H_2_O = 134:134:500. The amorphous cell module provides a variety of tools to build structures for any number of components at a chosen density and output a single structure [18,19]. These structures form the basic input to simulation and modeling workflows involving amorphous materials [20,21]. The thickness of the molecular model should sufficiently large to effectively exclude direct interactions between two different solution/solid interfaces created because of the periodicity of the system [22]. For systems containing a solid–fluid interface and dissolved solute, the distance between ions in the aqueous phase should not less than 8–10 Å [23]. Based on the molecular model of NaCl solution established by Kalinichev [22], the thickness of the solution layer is about 40 Å. Using the “Amorphous Cell” tool, a microscopic model of amorphous 8% NaCl solution is established in the range of 41.70 Å × 41.70 Å × 41.70 Å. After structural optimization, the stable molecular model of NaCl solution in Figure 2.

#### 3.1.3. Molecular Modeling of NaCl in the C-S-H Pore Structure

Minet [24] has experimentally determined that the pore width of C-S-H ranges from 0.5 nm to 10 nm. Based on the results obtained by Enrico Masoero [25], the predominant pore diameter of C-S-H is 3 nm when the hydration degree reaches 80%. Chloride ion transport causes deterioration of durability of RC structures during service. For cement composites, the hydration degree of cement in RC structure during the service period is usually about 80% [26]. Li [27] compared the attraction of C-S-H on chloride ion with different pore size. The results show that the C-S-H with smaller pore size has stronger adsorption effect on chloride ion. Therefore, C-S-H nanopores with pore sizes of 1.5 nm and 3 nm were selected for the molecular model.

**Figure 3:**
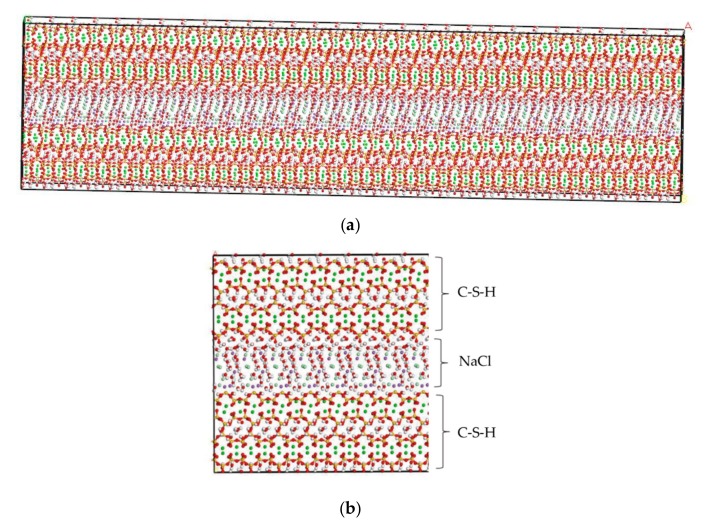
C-S-H pore structure model (green balls are Ca^2+^, yellow balls are Si atoms, purple balls are Na+, light green balls are Cl^−^, red balls are O atoms, white balls are H atoms). (**a**) Overall image. (**b**) Partial image.

By using MD simulation software, Hou proposed a method for testing ion diffusion coefficient under electric field [28]. In this study, the tools of “Cleave” and “Build Vacuum Slab” are employed to perform the molecular modeling of NaCl in the C-S-H pore structure [19,29,30]. The tool “Cleave” is employed to obtain the corresponding size model according to different needs, and the tool “Build Vacuum Slab” combines multiple types of models to form a composite model. Using the tools of “Cleave” and “Build Vacuum Slab”, a NaCl structure model with a size of 6.732 Å × 7.369 Å × 15 Å is obtained, in which case, the three designed facets, (1 0 0), (0 1 0), and (0 0 1) plans, of the NaCl solution model shown in Figure 2 are cut. By using the tool “Build Layer”, the C-S-H model and the NaCl solution model are then spliced into a C-S-H pore structure model with pore sizes of 1.5 nm and 3 nm, which is filled with 8% NaCl solution. The obtained model is a single cell structure which needs to be expanded to a supercell structure. To provide a better description of the C-S-H pore structure but without increasing extra computational efforts, a 30 × 2 × 1 super-cell structure is generated, which makes the simulation result closer to the real condition. The obtained initial structure is not stable, in which case, an optimization process must be performed to obtain a stable structure with the lowest energy. After optimization, a stable C-S-H pore structure is established, as shown in Figure 3. Using the Voigte–Reusse–Hill method [31], the calculated elastic modulus of C-S-H molecular model are 16.79 GPa, which is consistent with the experimentally obtained values (18.1 ± 4 GPa) of low-density C-S-H by Jennings [32].

For the C-S-H pore structure model without subject loading, the pore structure remains unchanged during the simulation process. Therefore, the MD simulation process of NaCl transportation in C-S-H nanopores is conducted in a canonical ensemble (NVT). All thermostatting schemes employed in MD simulations are designed to control the kinetic temperature [33]. The temperature was set at 298 K, which is controlled by the Nosé-Hoover method with a time step of 1 fs. The transport of NaCl in C-S-H nanopores with sizes of 1.5 nm and 3 nm is simulated with a time step of 6000 fs.

### 3.2. Fatigue Loading Application

**Figure 4:**
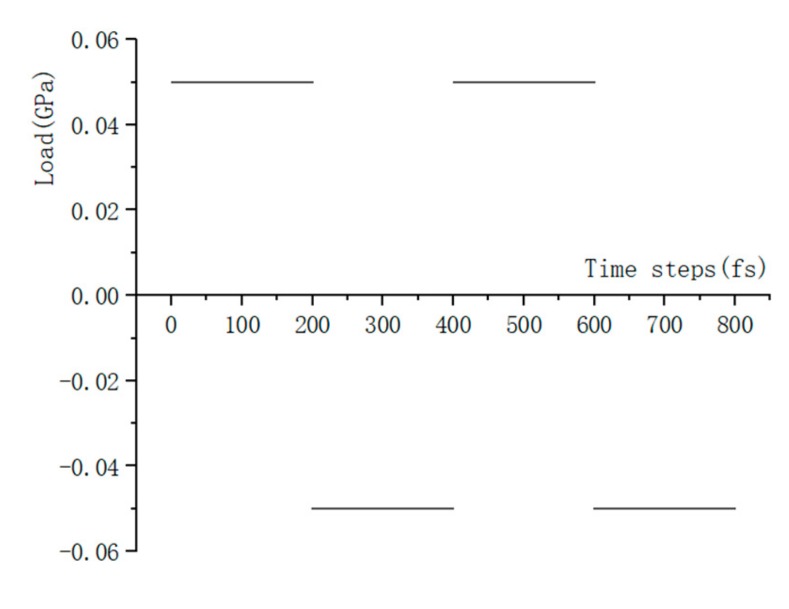
Loading process.

**Figure 5:**
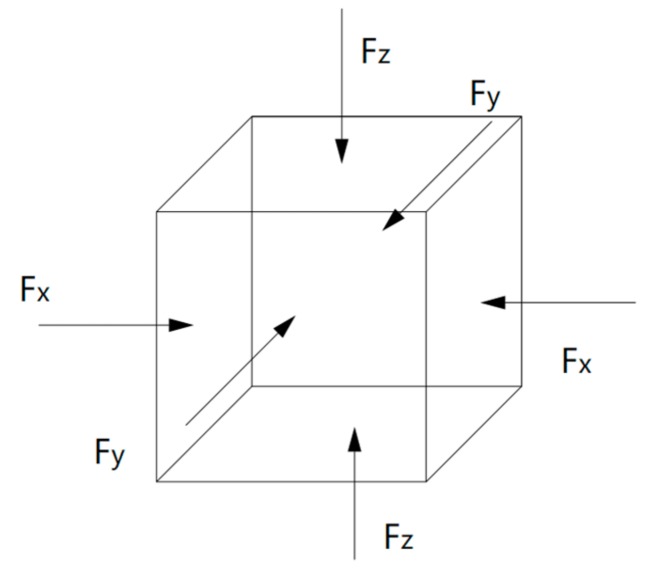
Schematic diagram of pressure application.

Compared to the model size of cement-based materials on the macro-scale, the size of the C-S-H molecular model on the macro-scale is far too small, and the fatigue loading cannot be applied directly to the molecular model of the C-S-H pore structure. Considering that the pore structure of C-S-H will change under fatigue loading, a periodic loading is applied along the *z*-axis direction for the molecular model constructed in Section 3.1.3. An isothermal-isobaric (NPT) ensemble is adopted in MD simulation, in which the volume of the structural system can change freely. The temperature is also set at 298 K, which is controlled by the Nosé–Hoover method. The periodic loading is controlled by the Parrinello–Rahman method [22], which can change the volume of the C-S-H pore structure. The MD simulation of NaCl transport properties in C-S-H nanopores with a size of 1.5 nm under periodic loading is conducted. Since the effect of external mechanical loading (compressive and tensile) on chloride diffusivity has been systematically investigated by Du [34]. In this study, the authors focus on the effect of fatigue loading on chloride diffusivity using MD simulation. During MD simulation, fatigue loading is regarded as sustained loading (compressive and tensile) that do not change for a period of time. The cyclic loadings are employed by considering the loading frequency and loading pattern [35,36]. The calculated diffusion coefficient depends on the loading process. Jiang [37] employed the number of fatigue cycles as the damage index which established the quantitative relationship between the fatigue damage degree and chloride diffusion coefficient. The results show that the diffusion coefficient of chloride ion in the structural concrete increases with the increase of loading frequency. In our investigation, only one loading frequency (0.25 MPa/fs) is adopted to examine the effect of fatigue loading on chloride diffusion in C-S-H pore structure. The adopted loading pattern is uniaxial alternate tension–compression fatigue loading (shown in Figure 4 and Figure 5), and the simulation is completed for 30 cycles in total.

### 3.3. Mean Square Displacement (MSD)

The MSD can be obtained directly from the particle positions in an MD simulation, as shown in Equation (3)

(3) $$MSD(\Delta t)=\frac{1}{T-\Delta t}\int_{0}^{T-\Delta t}{\left[r(t-\Delta t)-r(t)\right]}^{2}dt=\langle {\left[r(t-\Delta t)-r(t)\right]}^{2}\rangle$$

where T is the total simulation time; r(t) is the position at time t; r(t + Δt) is the position an interval Δt later; [r(t + Δt) − r(t)]^2^ is the squared displacement of the particle during that interval.

If the particle diffuses, the MSD becomes linear in time, and the slope defines the diffusion coefficient D_a_, as shown in Equation (4)

(4) $$D_{a}=\frac{1}{6N_{\alpha }}\underset{t\to \infty }{\lim}\frac{d}{dt}\sum_{t=1}^{N_{\alpha }}\langle \left[r_{i}(t)-r_{0}(t)\right]\rangle$$

where N is the number of diffusing atoms in the system.

## 4. Results and Discussion

### 4.1. Influence of Pore Size on NaCl Transport Properties in C-S-H Nanopores

**Figure 6:**
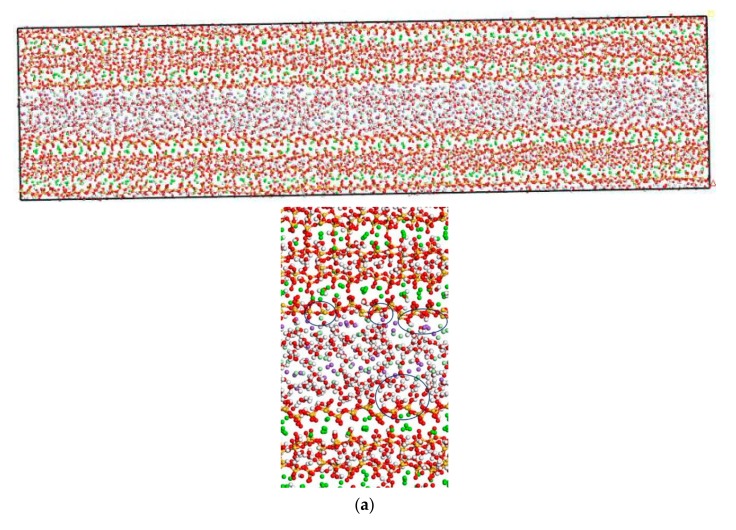

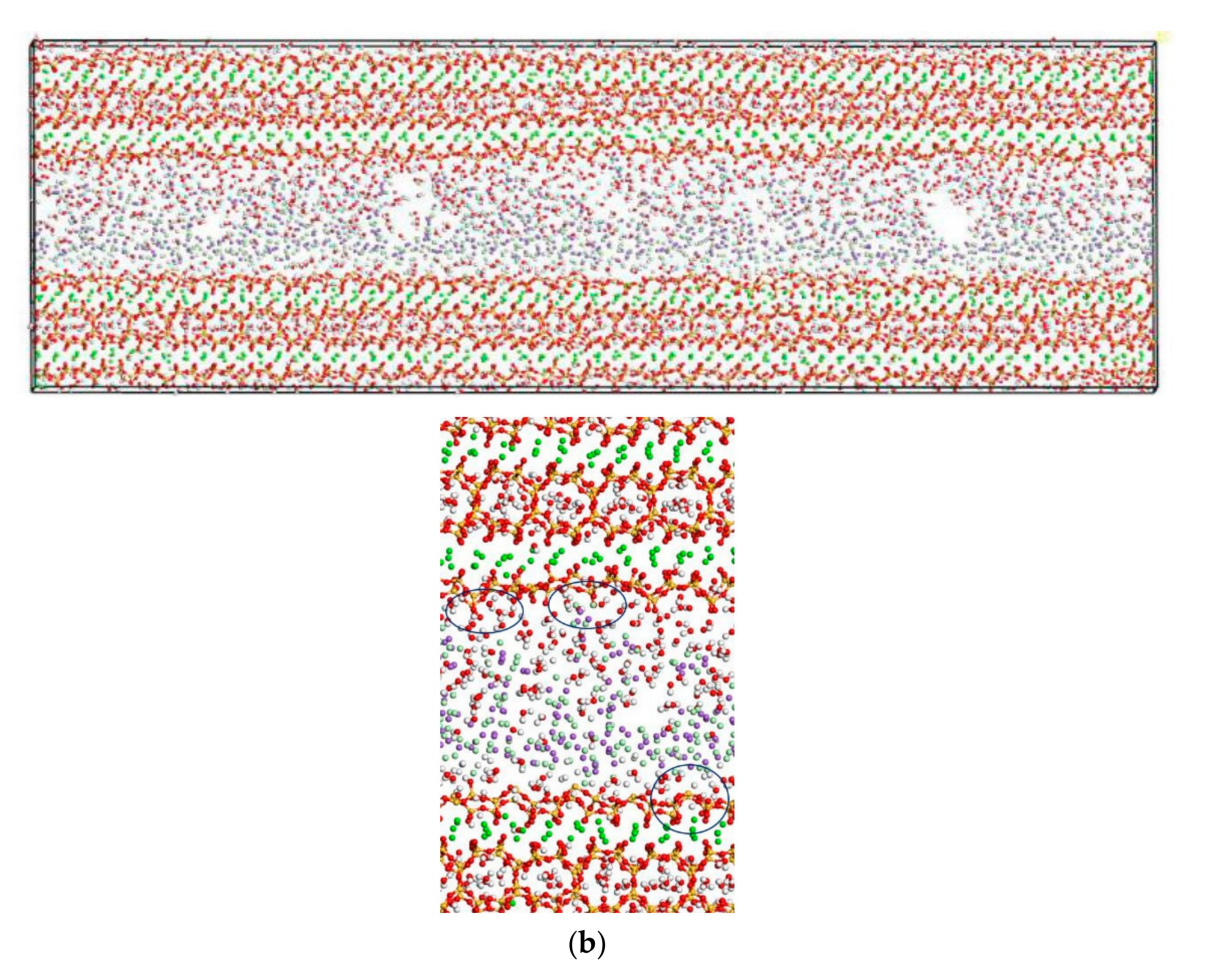
Transport properties of NaCl in C-S-H nanopores with different pore sizes (green balls are Ca^2+^, yellow balls are Si atoms, purple balls are Na^+^, light green balls are Cl^−^, red balls are O atoms, white balls are H atoms). (**a**) Pore diameter: 1.5 nm. (**b**) Pore diameter: 3 nm.

In this section, the transport properties of NaCl in C-S-H nanopores with different pore sizes and structural organization changes are investigated using the model constructed in Figure 3. Snapshots of molecules in the C-S-H pore structure after the completion of computation are shown in Figure 6. It is observed from the figure that the interlayer spacing of C-S-H is stable. Some chloride ions and sodium ions are transported to the wall of the pore structure. The number of chloride ions transported to the pore wall decreases with increasing pore size, which can be attributed to the interaction potential of the pore wall on chloride ions decreasing with increasing pore size [17].

### 4.2. Influence of Fatigue Loading on NaCl Transport Properties in C-S-H Nanopores

**Figure 7:**
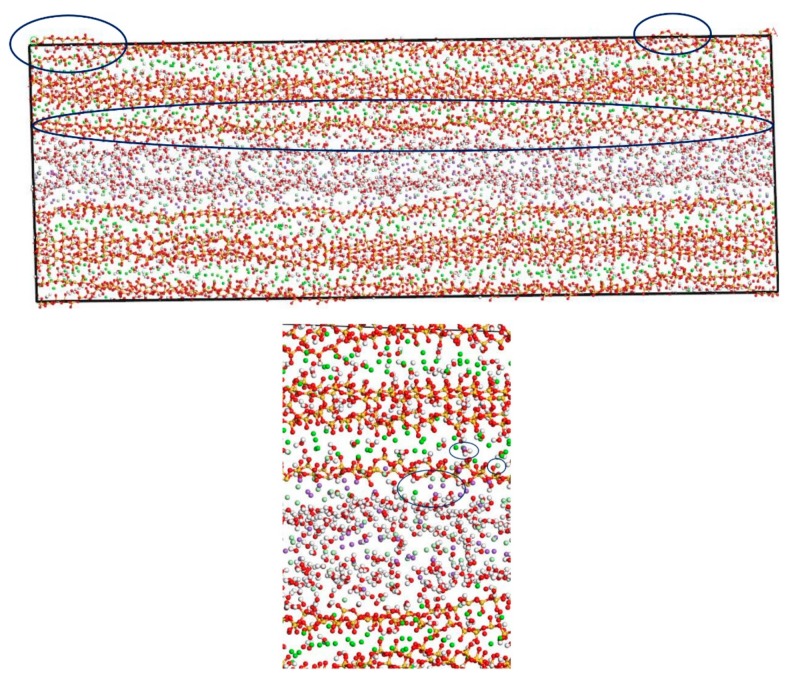
Transport properties of NaCl in C-S-H nanopores with pore size 1.5 nm under fatigue loading (green balls are Ca^2+^, yellow balls are Si atoms, purple balls are Na^+^, light green balls are Cl^−^, red balls are O atoms, white balls are H atoms).

In this section, the fatigue loading is exerted on the C-S-H pore structure with a pore size of 1.5 nm using the loading process shown in Figure 4. The transport properties of NaCl in C-S-H nanopores under fatigue loading are shown in Figure 7. It can be seen from the figure that the applied fatigue loading affects the C-S-H pore structure dramatically, leading to an increase in the interlayer spacing of C-S-H. The interlayer spacing increases from 2 Å to 3.5 Å, and lattice distortion occurs in part of the C-S-H pore structure. Some of the C-S-H is pulled out of the lattice range, and the pore structure is no longer a straight passage. It is also shown in Figure 7 that portions of Cl^−^ and Na^+^ are attracted by the pore wall, and part of the free Ca^2+^ leaves the C-S-H and enters the C-S-H nanopores.

Figure 6 and Figure 7 show that the amount of Na^+^ attracted to the surface of the C-S-H nanopores is higher than the amount of Cl^−^ attracted to the surface of C-S-H nanopores. Cl^−^ attracted to the pore wall surface is in the vicinity of Na^+^. The part of Cl^−,^ that enters the pore wall is close to Ca^2+,^ which indicates that the C-S-H pore wall provides strong adsorption for the cations. Na^+^ is attracted to the pore wall surface and combines with the silicon tetrahedron of the C-S-H structure. When the amount of combined Na^+^ increases, the effect of adsorption on Cl^−^ in the solution increases. Cl^−^ moves slowly towards the pore wall, which is adsorbed to the pore wall surface. Part of the Cl^−^ is attracted by the free Ca^2+^ in the pore wall structure and moves into the C-S-H nanopores.

**Figure 8:**
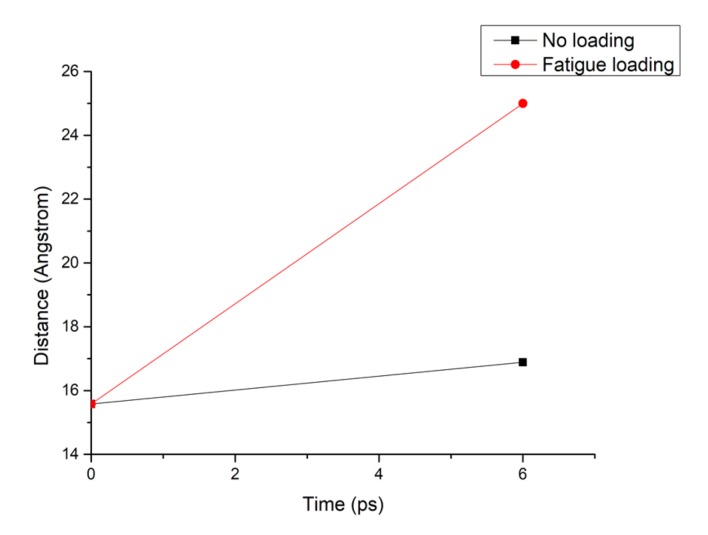
Pore diameter change of the C-S-H structure under different working conditions.

Figure 8 shows that the pore diameter of the C-S-H pore structure continuously increases with increasing simulation time. Compared with that of the C-S-H pore structure without subject fatigue loading, the diameter of C-S-H nanopores under fatigue loading increases approximately 7 times, which indicates that the C-S-H pore structure is continuously deformed by extrusion, leading to a looser structure and a wider pore size, thus affecting particle diffusion. With the increase of pore size, more particles will penetrate the concrete cover and diffuse to the surface of reinforcement, which leads to reinforcement corrosion.

### 4.3. Discussion

**Figure 9:**
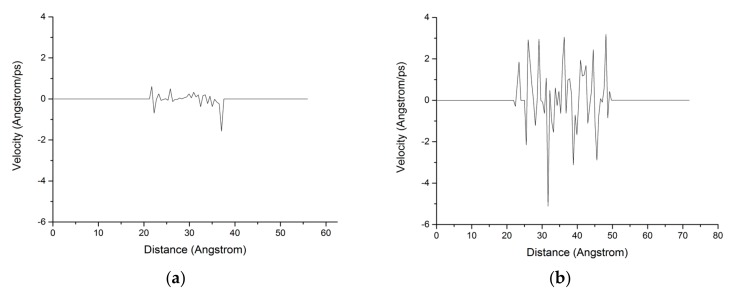
Distribution of the chloride ion diffusion rate in the C-S-H pore structure with a pore diameter of 1.5 nm. (**a**) Without fatigue loading; (**b**) Under fatigue loading.

The chloride ion diffusion rate perpendicular to the C-S-H nanopores with a diameter of 1.5 nm is calculated as shown in Figure 9. The figure shows that the chloride ion diffusion rate perpendicular to the C-S-H nanopores after the completion of fatigue loading is 4 times faster than that without fatigue loading. For the C-S-H nanopores without fatigue loading, Cl^−^ appears in the range of 20–40 Å. For the C-S-H nanopores under fatigue loading, Cl^−^ appears in the range of 20–50 Å, which indicates that the fatigue load forces a large amount of Cl^−^ into the pore wall. Ca^2+^ in the pore wall creates the potential for Cl^-^, which attracts Cl^−^ to move to the pore wall, thus affecting the chloride ion diffusion rate. As is shown in Figure 7, the application of loading leads to the weakness of the stiffness and cohesive force of C-S-H, in which case, different deflections and bending of calcium silicate sheet can be observed [38]. The discrepancy between interlayer connections results in the inhomogeneous diffusion of chloride ions in C-S-H pore structure.

**Figure 10:**
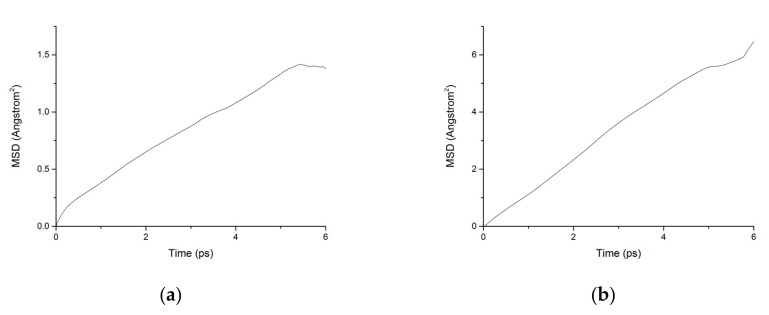
MSD diagram of water molecules in C-S-H nanopores without fatigue loading. (**a**) Pore diameter: 1.5 nm. (**b**) Pore diameter: 3 nm.

**Figure 11:**
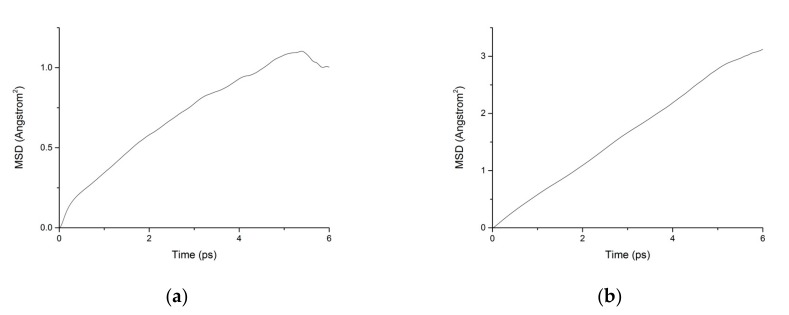
MSD diagram of chloride ions in C-S-H nanopores without fatigue loading. (**a**) Pore diameter: 1.5 nm. (**b**) Pore diameter: 3 nm.

**Figure 12:**
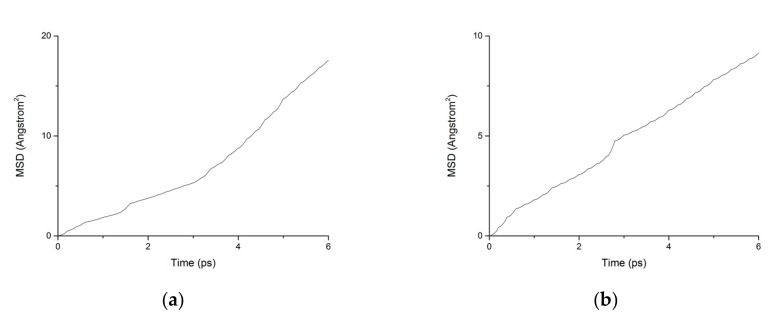
MSD diagram of particles in C-S-H nanopores under fatigue loading. (**a**) Water. (**b**) Chloride.

During the transport process of chloride ions, the transport characteristics of water molecules are particularly important for studying the transport characteristics of NaCl solutions in pore structures [39]. Therefore, the mean square displacements of water molecules and chloride ions in pore structures are displayed in Figure 10. The MSD of water molecules for both working condition are small, which indicates that the water molecules confined in the interlayer of C-S-H move slowly. The reasons can be attributed to the ionic bonds and H-bonds of C-S-H. The related diffusion coefficients under different conditions are calculated according to Figure 10, Figure 11 and Figure 12. The different trends shown in these figures at the time around 5 ps are attributed to the positive offset method adopted during the calculation [40,41]. The middle section of the curve is recommended for calculation to avoid errors [42]. The decrease of MSD curve is attributed to that the calculation of MSD is a function of time T instead of a function of time interval. As is shown in Table 2, the diffusion coefficient of water molecules is 1.794 × 10^−9^ m^2^/s when the pore diameter of the C-S-H nanopores is 3 nm. The diffusion coefficient of water molecules in nanopores can be experimental obtained by neutron scattering [43] and NMR [44]. Krynicki [23] measured the diffusion coefficient of water at temperatures between 275.2 and 498.2 K and at pressures up to 1.75 kbar, the measured value is (2.3 ± 0.1) × 10^−9^ m^2^/s, which is slightly higher than the simulation result. This is attributed to the adopted pore diameter of C-S-H nanopores in the MD simulation being 3 nm. For the measured cement-based materials, the pore diameter of the C-S-H nanopores ranged from 0.5 nm to 10 nm. The diffusion coefficient of the particles obtained by the experiment is the result of the particles being transported in a large number of pores with different pore sizes.

It is also shown in Table 2 that the diffusion coefficient of water molecules and chloride ions increases with the increased pore diameter of C-S-H nanopores. This rapid increase in the diffusion coefficient is attributed to the potential of the C-S-H pore wall for water molecules and chloride ions. As the C-S-H pore size increases, the effect of the interatomic potentials on the internal particles decreases, resulting in an increase in the diffusion coefficient of the particles.

Fatigue loading has a significant influence on the particle transport process. The pore diameter of C-S-H nanopores increases continuously under fatigue loading, which causes a decrease in the interatomic potentials on the particles in C-S-H pore structures, thus further increasing the diffusion coefficient of chloride ions and water molecules in the C-S-H structure. Moreover, since the C-S-H nanopores are constantly subjected to fatigue loading, the solution in the nanopores is continuously extruded, in which case, the transportation speed of the particles in the nanopores is greatly increased on the microscopic scale. Due to the influence of the various factors mentioned above, the diffusion coefficient of the particles in C-S-H nanopores is greatly increased.

As mentioned before, the diffusion coefficient obtained from MD simulation is different from the effective diffusion coefficient of C-S-H. However, MD simulation can get the results which is hard to be obtained by experiments. Furthermore, MD simulation can reveal the essence of transport process of particles in cement composites, which can add significant additional molecular scale insight to experimental results for structural concrete.

## 5. Conclusions

In this paper, the molecular models of C-S-H, NaCl solution, and C-S-H nanopores containing NaCl are established using MD simulation software. The influence of pore size and fatigue loading on the transport properties of NaCl in C-S-H nanopores were investigated. The main findings are summarized below:

(1) Ca^2+^ and Na^+^ in the pore wall of C-S-H nanopores can adsorb chloride ions, attracting chloride ions to move towards the pore wall. The attraction effect varies with the pore diameter of C-S-H nanopores. The larger the pore diameter is, the weaker the attractive interaction of the pore walls to chloride ions. The C-S-H pore wall had a strong adsorption effect for cations. The amount of Cl^−^ adsorbed in the solution increases with the increase of the amount of Na^+^ adsorbed on the pore wall, in which case, chloride ions will be adsorbed to the surface of the pore wall of C-S-H nanopores. In addition, some Cl^−^ will be adsorbed by free Ca^2+^ in the pore wall of C-S-H nanopores and move to the interior of the C-S-H pore structure.
(2) The C-S-H pore structure is continuously deformed by extrusion under fatigue loading, which leads to the expansion of pore size, thus increasing the chloride diffusion rate. At the same time, more chloride ions will penetrate the concrete cover and diffuse to the surface of reinforcement, which leads to a great increase in the corrosion probability of reinforcement and a decrease in the durability of reinforced concrete structure.
(3) The diffusion coefficient of water molecules in C-S-H nanopores with a pore size of 3 nm obtained from the MD simulation is 1.794 × 10^−9^ m^2^/s, which is slightly lower than that obtained from the experiment. This is attributed to the diffusion coefficient of the particles obtained by the experiment being the result of the particles being transported in a large number of pores with different pore sizes.
(4) In this paper, only the transmission process of chloride ions in a single nanopore is considered. The concrete composites contain variety of pore structures with different size. A molecular model with different pore sizes and structures should be established, which can further improve the accuracy of numerical simulation.

## Figures and Tables

**Table 1:** Lattice parameters of tobermorite 11 Å

Category	Tobermorite 11 Å
Space group	Bm
Crystal system	Monoclinic
a/Å	6.732
b/Å	7.369
c/Å	22.68
α/°	90
β/°	90
γ/°	123.18

**Table 2:** Diffusion coefficient of water molecules and chloride ions under different working conditions

Diffusion Coefficient (×10^−9^ m^2^/s)	Without Fatigue Loading		Under Fatigue Loading
	Pore Diameter: 1.5 nm	Pore Diameter: 3.0 nm	
Chloride ion	0.287	0.896	2.502
Water molecule	0.383	1.794	4.779
